# The contribution of Swiss scientists to the assessment of energy metabolism

**DOI:** 10.1038/s41430-018-0139-5

**Published:** 2018-05-10

**Authors:** Jean-Pierre Montani, Yves Schutz, Abdul G. Dulloo

**Affiliations:** Division of Physiology, Faculty of Sciences and Medicine, University of Fribourg, Fribourg, Switzerland

## Abstract

Although Switzerland is considered a small country, it has its share in discoveries, inventions and developments for the assessment of energy metabolism. This includes seminal contributions to respiratory and metabolic physiology and to devices for measuring energy expenditure by direct and indirect calorimetry in vivo in humans and small animals (as well as in vitro in organs/tissues), for the purpose of evaluating the basic nutritional requirements. A strong momentum came during World War II when it was necessary to evaluate the energy requirements of soldiers protecting the country by assessing their energy expenditure, as well as to determine the nutritional needs of the Swiss civil population in time of war when food rationing was necessary to ensure national neutrality and independence. A further impetus came in the 1970s at the start of the obesity epidemics, toward a better understanding of the metabolic basis of obesity, ranging from the development of whole-body concepts to molecular mechanisms. In a trip down memory lane, this review focuses on some of the earlier leading Swiss scientists who have contributed to a better understanding of the field.

## Introduction

The assessment of energy metabolism is crucial to the understanding of basic nutritional requirements during growth, in time of famine and food scarcity, as well as during times of food excess for a proper control of body weight. Since the first experiments in the 1780s by the French scientists Antoine Lavoisier and Simon de Laplace to show that heat production by animals can be predicted from oxygen consumption, many scientists all over the world have contributed to a better understanding of energy metabolism and methods to assess it. The purpose of the current review is to highlight the contribution of leading Swiss scientists who have helped in this endeavor. We are limiting our review to Swiss scientists who have left us or who are no longer active in research on energy metabolism.

## The history of brown adipose tissue (BAT) begins in 1551

**Fig. 1:**
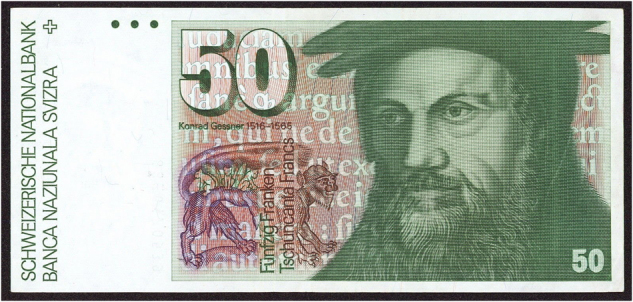
Fifty francs Swiss banknote, from the sixth series of Swiss banknotes, issued between 1978 and 2000 showing Konrad Gessner (1516–1565) who was the precursor of brown fat description

The history of Swiss scientists who have contributed to the understanding of energy metabolism in animals may have started with Konrad Gessner (1516–1565), a physician, botanist, zoologist and bibliographer. Born in Zurich in a family of eight children with little means, he spent much of his childhood with his great uncle Johannes Frick, a chaplain and collector of medicinal herbs, who instilled in him a lifelong interest in natural history. In his great zoological work, *Historiae animalium*, a collection of four volumes published in medieval Latin between 1551 and 1558 and later translated in German, he described with careful observations and often dissection many living animals, quadrupeds, amphibians, birds and fishes. In the first volume devoted to viviparous quadrupeds (*De Quadrupedibus vivipares*, 1551), he writes on page 842 that the marmots, although quite meagre in all body parts, have a very fat back, but this tissue was “neither fat, nor flesh [*nec pinguitudo, nec caro*]—but rather something in between” [1]. This tissue turned out to be BAT, a tissue that is neither muscle (flesh), nor white adipose tissue. Gessner’s contributions to sciences were honored on the Swiss fifty-franc banknote, issued between 1978 and 2000 (Fig. 1). After the life of Gessner, not much is noted in the field of energy metabolism in Switzerland over the subsequent three and a half centuries, in contrast to surrounding countries such as France with Antoine Lavoisier. Yet there were important contributions to physiological sciences with a strong interest in respiratory and high-altitude physiology throughout the eighteenth and nineteenth centuries.

## The scandal of 1st August 1917

1st August is the Swiss National Day, established in 1891 by the Federal Council to commemorate the 600th anniversary of the foundation of Switzerland in 1291, in the fight for independence against the House of Habsburg. National guidelines were to ring all churches’ bells at 7 p.m. on that day throughout the country, followed later in the evening by bonfires on higher grounds. However, on that day of 1917, in the midst of World War I, the bells of the protestant church of Wipkingen, a suburb of Zurich, remained silent [2]. In not ringing the bells, the church minister, Ernst Altwegg, created a scandal to show his support in favor of a conscientious objector; a young 24-year old lieutenant in the Swiss army and brilliant student at the Swiss Federal Institute of Technology, who decided in spring of the same year not to serve anymore. For that daring decision, he was expelled from the Institute, spent 4 months in prison and was stripped of civil rights for 1 year, a decision approved by the Federal Council.

This student was Max Kleiber (1893–1976), born and educated in Zurich, very much interested from a young age in agriculture and animal nutritional needs. However, thanks to the support of academicians, he was allowed to reintegrate into the Federal Institute of Technology and graduate in 1920 as an agricultural chemist, to later obtain his PhD in 1924 and the Privat-Docent title in 1927 from the same school. In 1929, at the age of 36, he moved to the United States to become professor in the Animal Husbandry Department at the University of California at Davis, “to construct respiration chambers and conduct research on energy metabolism in animals” [3] and where he stayed until his retirement.

## Max Kleiber and the fire of life

**Fig. 2:**
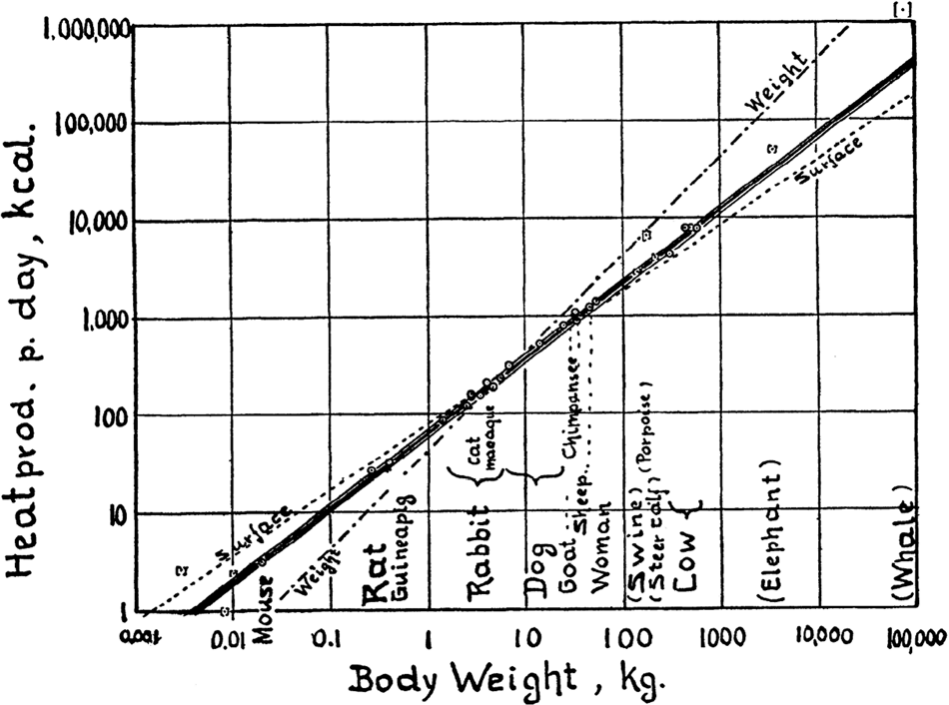
Log of metabolic rate vs log of body weight from shrew to whale, from Kleiber [7]

In 1961, Max Kleiber published the Fire of Life [4], a fundamental monography, which summarizes many concepts in animal metabolism, based on his own observations in Switzerland and later in the United States and on data collected by other researchers in the field. A second and last edition followed in 1975 [5]. A first concept published by Kleiber in 1932 [6] was that the body weight to the power of three-fourths (W^3/4^) was more reliable than just body weight or body surface area to predict the basal metabolic rate of animals across species and to compare energy requirements among animals of different sizes across species. The initial graph from 1932 spanned from the mouse to the cow. This graph was then extended in the Physiological Review of 1947 [7] from the shrew to the whale in a handmade plot shown in Fig. 2. However, computation for metabolic rate prediction was still made from a 21 g mouse to a 600 kg cow and did not include smaller (such as 10.5 g Swiss mice or a 3.5 g shrew) or larger animals (such a 3.6 tons elephant or a 70 tons whale). Reasons for not including these extreme animals, who were outside the fitting line (see Fig. 2), are carefully given in the review: for the shrew because based on one single measurement, for the Swiss mice for not being in the post-absorptive state. For larger animals, “the conditions of measurement of metabolic rate of elephant, porpoise and whale are not strictly in line with the normal conditions of measuring standard metabolic rates” (cited from Kleiber [7], page 528).

Another concept well studied by Kleiber was the relation between resting (basal) energy expenditure (EE) and environmental temperature, with a zone known as thermoneutral zone with minimal EE, which varies depending of the species, rabbit, dog, rat and hairless mice (Fire of Life, 1961, page 162). Outside of this zone, EE increases both at higher and lower temperatures. Max Kleiber also pioneered the use of isotopes to study metabolic processes in the living animal, particularly in dairy cow chosen for its agricultural importance [8]. Advances in isotope technology and issues of thermoneutrality are still nowadays hot topics of contemporary research.

## Max Kleiber and Albert Einstein: common destinies

There is some interesting parallelism between the lives of Max Kleiber and Albert Einstein (1879–1955). Both were interested in energy, atomic for one, metabolic for the other one. In fact, both are known for very simple but fundamental formulas, E = mc^2^ for Einstein and M = ~W^3/4^ for Kleiber (where M is the metabolic rate and W the body weight of the animal). Both studied, graduated and taught at the Swiss Federal Institute of Technology in Zurich. Both objected to military service and remained pacifists all their lives. If Kleiber became an objector of conscience at the age of 24, Einstein renounced his citizenship of the German Kingdom of Württemberg at the age of 17 to avoid military service [9]; he gained the Swiss citizenship in 1901, at the age of 22, and kept it until his death in 1955. Both emigrated to the United States, Kleiber in 1929 at the age of 36 and Einstein in 1933 at the age of 54, and they both acquired the US citizenship, Kleiber in 1939 and Einstein in 1940.

## Alfred Fleisch, the inventor

Alfred Fleisch (1892–1973) was born and schooled in Dietikon near Zurich. He later studied medicine at the University of Zurich (1911–1917). His passion for physiology grew very strong over the years, showing in his thesis of Doctor in Medicine defended in 1918 that carbonic acid dilates blood vessels. The biographical details of Alfred Fleisch have been summarized in the review of Peet-Henn Kingisepp [10], inspired by the years spent by Fleisch as professor of Physiology at the University of Tartu in Estonia. In 1932, Fleisch took the Chair of the Institute of Physiology at the University of Lausanne where he worked and taught until his retirement in 1962.

Alfred Fleisch invented several medical devices that helped considerably in the study of respiratory physiology and energy metabolism. Three devices stand out particularly:

**(1)** The pneumotachograph [11] is a device for measurement of instantaneous air flow at the mouth, a device crucial for the understanding of the kinetics of O_2_ consumption and CO_2_ production. Before that time, O_2_ consumption was primarily measured from the rate of decrease of the air chamber in a closed-circuit water-sealed spirometer containing a CO_2_ absorber. Since all the CO_2_ exhaled in the spirometer was absorbed, any decrease of the volume in the air chamber could be attributed to the disappearance of oxygen. That system was quite bulky and inadequate for the assessment of rapid changes in O_2_ consumption; furthermore, it did not allow the simultaneous measurement of CO_2_ production for computation of respiratory quotient, a ratio essential to assess fat versus carbohydrate oxidation rates.

**Fig. 3:**
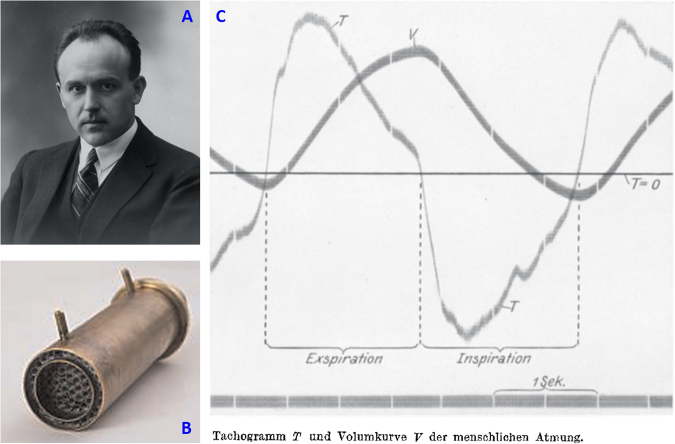
**a** Alfred Fleisch. **b** Pneumotachograph: pictures **a** and **b** from Kingisepp [10]. **c** Original recording of flow (Tachogramm (T)) and volume (V) at the mouth with the pneumotachograph, from Fleisch [11]

The genius of Alfred Fleisch was based on simple physics of dynamic air flow stating that the drop in air pressure across a tube (mouth piece) with fixed resistance ensured by a fine lamellar grid would be directly proportional to air flow. Integrating flow would then yield volume values (Fig. 3). That device, central in modern spirometry for assessment of pulmonary volumes and determination of flow-volume loop curves during forced expiration, could also be used, knowing the fraction of exhaled O_2_ and CO_2_ during normal breathing, for measurement of continuous O_2_ consumption and CO_2_ production.

**(2)** The ergostat [12] was a new type of friction-loaded ergometer that solved a major limitation of classical friction-based ergometers in which resistance to cycling was ensured by the area of surface contact and pressure of a belt against a rotating wheel. Indeed, as the subject was pedaling for longer times or at increasing pedaling rates, the classical belt would heat up, which would change the frictional forces. In addition, it was very difficult to set a given braking torque. In a clever design fully described in the review by Vandewalle and Driss [13], the addition of a loose rotating wheel controlled by a rope to which a pre-set weight was attached allowed to compensate for any change in time of the frictional forces. In addition, it was easy to set the device very precisely at the desired braking torque by playing with the weights attached to the running and the loose wheels. This ergometer is still used in many laboratories of exercise physiology.

**(3)** The metabograph [14, 15] was a bulky apparatus developed for continuous online measurements of minute ventilation, O_2_ consumption and CO_2_ production. The apparatus was complex with many tubes and pumps, chemical titration of KOH for CO_2_ determination and required a heavy maintenance. A detailed description of the functioning of Fleisch’s metabograph is available from the Website (https://www.pftforum.com/history/fleisch-metabograph-diagram-1963/). Despite its complexity, the system was very useful to follow the kinetics of gas exchange, such as during an exercise load.

## Albert Fleisch and food rationing in war times

During World War I, Switzerland experienced a crisis in food supply worsened by a bad preparation to war economy. Food rationing was only introduced in March 1917, first for rice and sugar, and later extended to cereals, dairy products, oil and other commodities. However, this could not really prevent some moderate undernourishment of the population in 1918 [16]. With the advent of World War II, Switzerland was better prepared. A Federal Office of Alimentation had already been created in September 1918 and a State structure to manage war economy was fully in place by 1938. However, before the war only 50% of the caloric needs of the population could be covered by internal production. Under the direction of the agricultural engineer Friedrich Traugott Wahlen (1899–1985), chief of the Division of Agriculture Production and Domestic Economy at the Federal Office of Alimentation, a strategic plan called the “Wahlen Plan” was developed. Its purpose was to increase agriculture land surface by changing lawns and prairies into agricultural fields, in order to be able to become self-sufficient in case all food importations would stop [17]. This enabled Switzerland to cover domestic needs for potatoes, fruits and vegetables, but the country still had to rely on importations for many other food products. As importations became harder with time, strict food rationing was introduced. The task to evaluate the nutritional needs of the population was given to the Federal Committee for wartime feeding (*Commission fédérale pour l’alimentation de guerre, CFAG*) under the presidency of Alfred Fleisch.

**Fig. 4:**
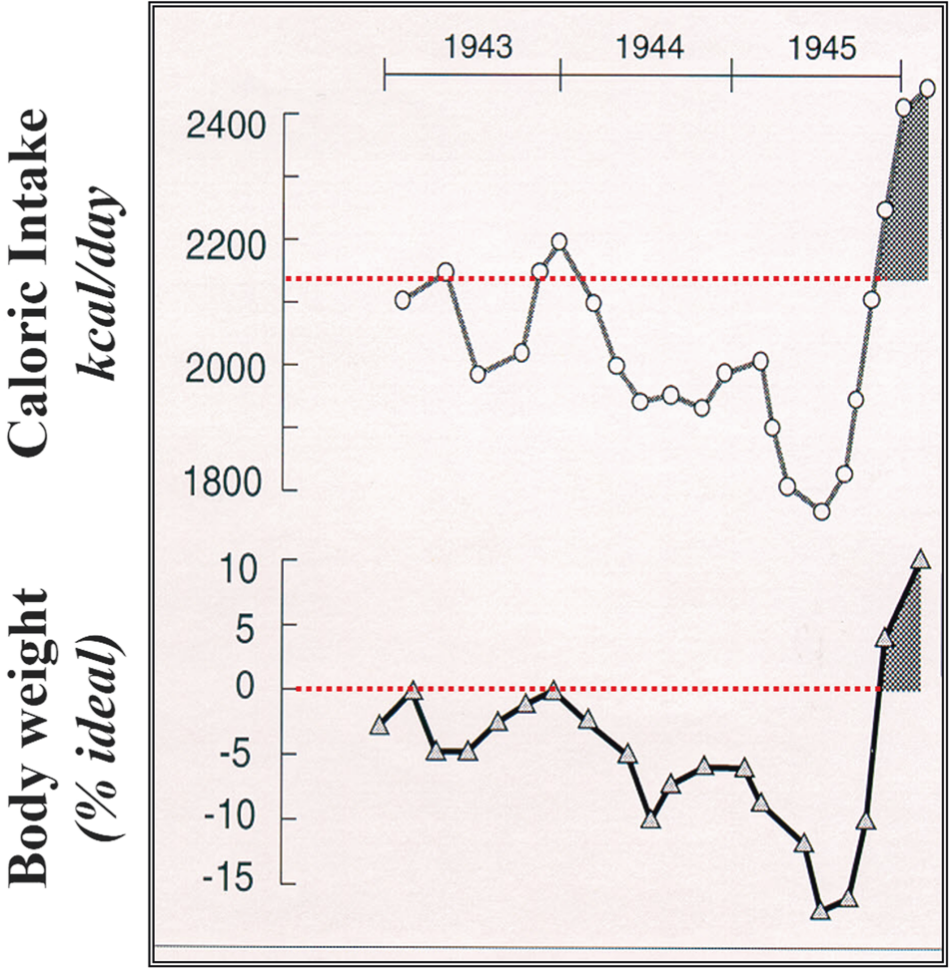
Average caloric intake fluctuation during World War II restriction in Switzerland, showing the effect on body weight change (as percentage of ideal weight). Figure drawn from Fleisch [19]

The Committee had to establish, based on scientific evidences, the caloric needs and the amounts of milk, proteins from eggs and meat, fats and vitamins for various groups of population including children, soldiers and heavy workers, for the determination of food coupons. This was also the opportunity for Alfred Fleisch to follow 700 persons of various ages and social conditions from autumn 1941 to spring 1946, by monitoring body weight, hemoglobin, work conditions and food intake, as well as the occurrence of various diseases [18]. As shown in Fig. 4 drawn using data from the 1947 book of Fleisch [19], the recommended caloric intake of 2160 kcal/day for an adult not doing extra physical work could not be maintained already from 1943, with seasonal shortages in intake leading to seasonal variations in body weight and a worsening over the following years. Interestingly, as rationing could be progressively stopped after summer 1945, there was a period of hyperphagia resulting in an excess of body weight—a phenomenon of caloric overcompensation and weight overshooting that was also observed in the classic Minnesota Starvation Experiment conducted in the United States at the end of World War II [20].

## The post-war era and the emergence of diseases of affluence

In the few decades following World War II, the industrialized nations were facing the challenges of the “diseases of civilization” arising from the surge in mechanization and technology in everyday life. This not only led to drastic reductions in physical activity, but also easy access to a modern “Western” diet consisting of refined, energy-dense foods rich in fat, sugars and salt. With firm links established between diet and disease, such as the classic “Seven Country Study” linking diet and heart diseases [21], understanding “how much food does man require” took a much larger significance than simply coping with the minimum for survival in times of war and famine. The central theme was how to explain the large inter-individual variability in human energy requirements as revealed in studies conducted between 1945–1970 and embodied in a 1973 paper in Nature [22] entitled “*How much food does man require?*” The key points of this “opinion paper” authored by four eminent British nutritionists are: (1) the energy requirements of man and his balance of intake and expenditure are not known; (2) there are subjects who have been given large quantities of additional food with little or no increase in body weight; (3) obese subjects experience difficulties in reducing body weight despite drastic reductions of food intake; and (4) many obese people seemed to eat no more, and sometimes even less, than those who are not obese. The authors concluded that “*these observations underline the extent of our ignorance about the mechanisms by which energy balance is maintained*”.

Although emphasis was put on the lack of understanding of the metabolic differences that must lie behind inter-individual variabilities in energy needs, it was also increasingly recognized that because of potentially large errors in the assessment of food intake (including its underreporting), human energy requirements under various conditions should be studied by measuring EE. This recommendation was taken up by the Joint FAO/WHO Ad Hoc Expert Committee (1985) on “Energy and protein requirement” [23], which stated that “*As a matter of principle, we believe that estimates of energy requirements should, as far as possible, be based on estimates of energy expenditure, whether actual or desirable*”.

Concomitantly, the early 1970s saw the emergence of a new generation of Swiss scientists in the field of energy metabolism whose work in the development and application of various calorimetry systems took center stage in addressing many issues toward understanding how body weight is regulated. The development of calorimetric tools was already appreciated by the expert scientists of the Marcel Benoist Foundation.

## The 1973 laureates of the Marcel Benoist Swiss Science Prize

Marcel Benoist, a Frenchman living in Switzerland at the beginning of the 20th century, was a great humanist. At his death in 1918, he left his financial assets to the Swiss Confederation. In his will, he indicated that “revenue from these assets shall be used to award annually a single prize to a Swiss scholar or a scholar resident in Switzerland who in the course of that year has made the most useful discovery or study in the sciences that is of particular relevance to human life” [24]. Since 1920, the Marcel Benoist Foundation has awarded annually the Marcel Benoist Swiss Science Prize, a prestigious prize also known as the “Swiss Nobel Prize”. So far, 10 Marcel Benoist Swiss Science Prize laureates went on to receive the Nobel Prize—10% of the total [25].

**Fig. 5:**
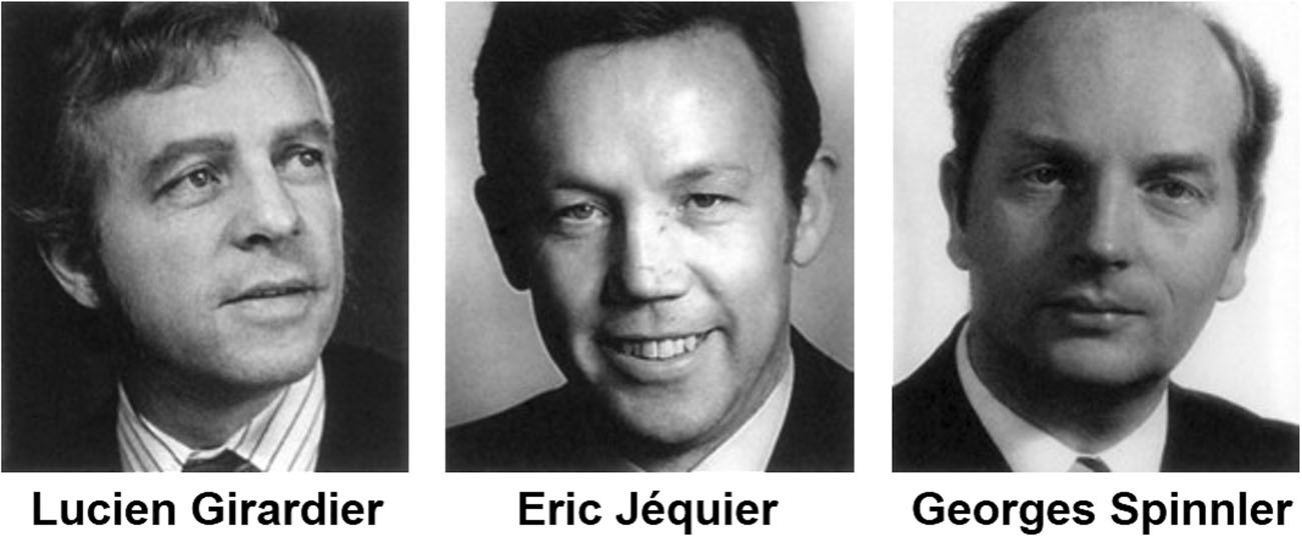
The 1973 laureates of the prestigious national award in Science, the “Marcel Benoist Swiss Science Prize”. Source: https://marcel-benoist.ch/en/current-laureate/past-laureates/ (accessed 20 Dec 2017)

In 1973, the laureates of the Prize were Eric Jéquier, Professor and Head of Physiology at the University of Lausanne, Lucien Girardier, Professor of Physiology at the University of Geneva and Georges Spinnler, Professor of Mechanics at Swiss Federal Institute of Technology in Lausanne (EPFL) (Fig. 5). They were awarded the prize “*for their work on the development of direct and indirect microcalorimetry and macrocalorimetry, and their application to human physiopathology and basic research*” [26], which shows the common interests of these three schools for the assessment of energy metabolism in both humans and animals as well as tissue/organ level.

## The school of Lausanne

### Thermal balance and temperature regulation

The contribution of Lausanne to the assessment of energy metabolism can be traced to the work of Eric Jéquier and collaborators in the early 1970s when they were applying direct calorimetry to study various aspects of thermal balance and temperature regulation in newborns and in adults, in particular: (i) the relationship between body size, thermal balance and thermal insulation in the newborn infants under various ambient conditions (including relative humidity, and heat exposure) [27]; (ii) the regulation of heat loss during muscular work and exercise in adults [28]; and (iii) the impact of a greater fat insulation upon the regulation of heat loss by comparing lean and subjects with varying degrees of obesity [29].

**Fig. 6:**
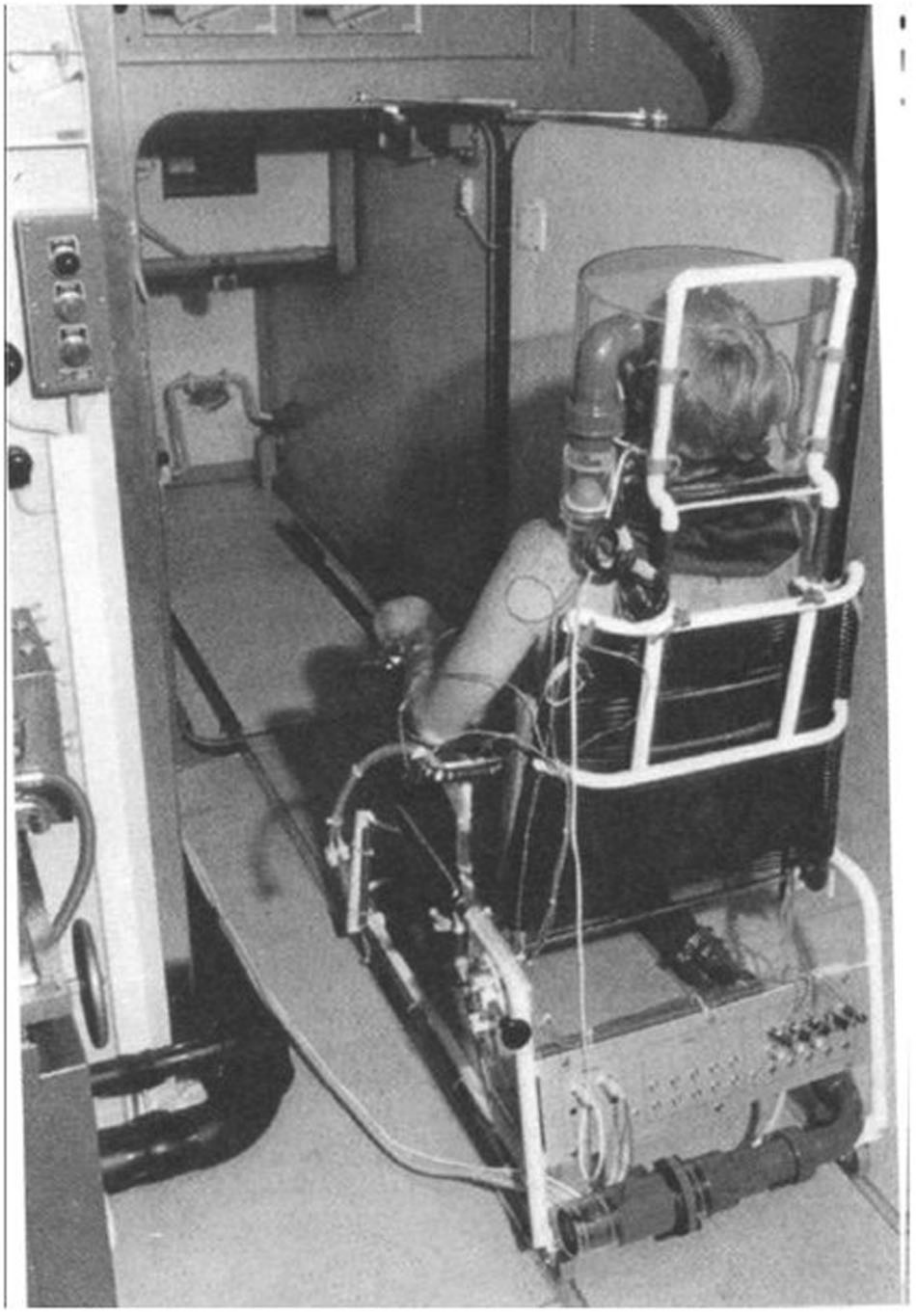
Photograph of the gradient layer direct calorimeter (size 1.56 m^3^) coupled with indirect calorimetry with a ventilated hood system (courtesy of Eric Ravussin)

A key development in this line of investigation was the construction in Lausanne of a gradient layer direct calorimeter, in collaboration with Georges Spinnler [30]. This method not only measured total heat losses but separated the measurements of dry heat loss (radiative and convective) and evaporative heat loss (insensible perspiration and sweating). It could be coupled with an open-circuit indirect calorimetry system with a ventilated hood (Fig. 6).

This approach revealed that moderately overweight and obese subjects showed lower heat losses and higher thermal body insulation than controls, leading the authors to conclude that “*the decreased heat loss may contribute to their positive energy balance by decreasing the energy cost of maintaining body temperature during cold exposure*”. Some 35 years later, this notion that diminished cost of homeothermy may predispose (or perpetuate) obesity was revitalized by Lewis Landsberg [31] and this area of research has since gained momentum with the availability of ingestible pill size telemetry sensors that allows continuous measurement of core body temperature over the 24-h cycle.

### Thermic effects of nutrients and substrate oxidation

By mid-1970s, the interest in the application of direct calorimetry to study thermoregulation in obesity extended to the application of indirect calorimetry (using an open-circuit ventilated hood system) to study both EE and respiratory quotient (which provides an indication of the proportion of EE derived from fat and carbohydrate oxidation).

A first objective was to reinvestigate the classical concept of “specific dynamic action” of food—that is, the thermic effect of nutrients—at a time when results in human subjects were scarce in the literature. This contributed to the currently accepted values of thermic effect of nutrients expressed as percentage of their energy content, that is, 2–3% for lipids, 6–8% for carbohydrates and 25–30% for proteins [32]. It also led to the concept of partitioning of the thermic effect of nutriments into two separate components that can be described as “obligatory” and “regulatory” thermogenesis, as demonstrated for glucose [33]. The former is due to the energy costs of digesting, absorbing and converting the nutrients to their respective storage forms. The latter is an energy dissipative mechanism of potential relevance to a defect in thermogenesis contributing to the development of obesity and involves the actions of insulin and the autonomic nervous system [33–35]. Furthermore, a blunted glucose-induced thermogenesis in obese before or after weight loss was thought to contribute to the relapse of obesity [36, 37].

A second objective was to study carbohydrate and lipid metabolism in lean subjects, in obese and diabetic patients. In collaboration with Jean-Pierre Felber (1922–2013), this was achieved by indirect calorimetry with the ventilated hood system often coupled with the euglycaemic insulin clamp technique to assess insulin sensitivity. Those experiments showed that the main effect of insulin on carbohydrate metabolism is to stimulate glucose storage, and that impairment of glucose storage, in particular by excess lipids, is a major defect of glucose utilization in type 2 diabetes [38, 39]. Furthermore, in studies conducted in collaboration with Albert Burger, the use of indirect calorimetry coupled to euglycemic insulin clamp techniques provided important insights in the glucoregulatory function of thyroid hormones, as well as the role of endogenous insulin in the effects of thyroid hormones on thermogenesis and in the control of glucose and lipid metabolism [40, 41].

A third objective, also in collaboration with Jean-Pierre Felber, was to test the efficacy of novel candidate drugs [42] in stimulation thermogenesis and the potential impact on substrate oxidation and glycemic control—an active area of research following the discovery of a new beta adrenoceptor (beta-3) and interest by several pharmaceutical houses (including nearby Hoffman La Roche in Basel) in developing beta-3 agonists for treating obesity. In addition, the thermogenic response to catecholamines was evaluated in acute clinical studies in healthy men [43, 44].

A fourth objective was to gain insights into the mechanisms by which altered sympathetic nervous system activity (often elevated in obesity) may play a role in the complications of obesity. In collaboration with Urs Scherrer, they performed studies involving simultaneous measurements of muscle sympathetic nerve activity and calf muscle blood flow together with carbohydrate oxidation rate during hyperinsulinemic euglycemic clamp. Notably, they demonstrated that insulin resistance in obese subjects is associated with increased fasting skeletal muscle sympathetic activity together with a specific impairment of sympathetic neural responsiveness to hyperinsulinemia in this tissue [45].

### The importance of 24-h indirect calorimetry chamber

**Fig. 7:**
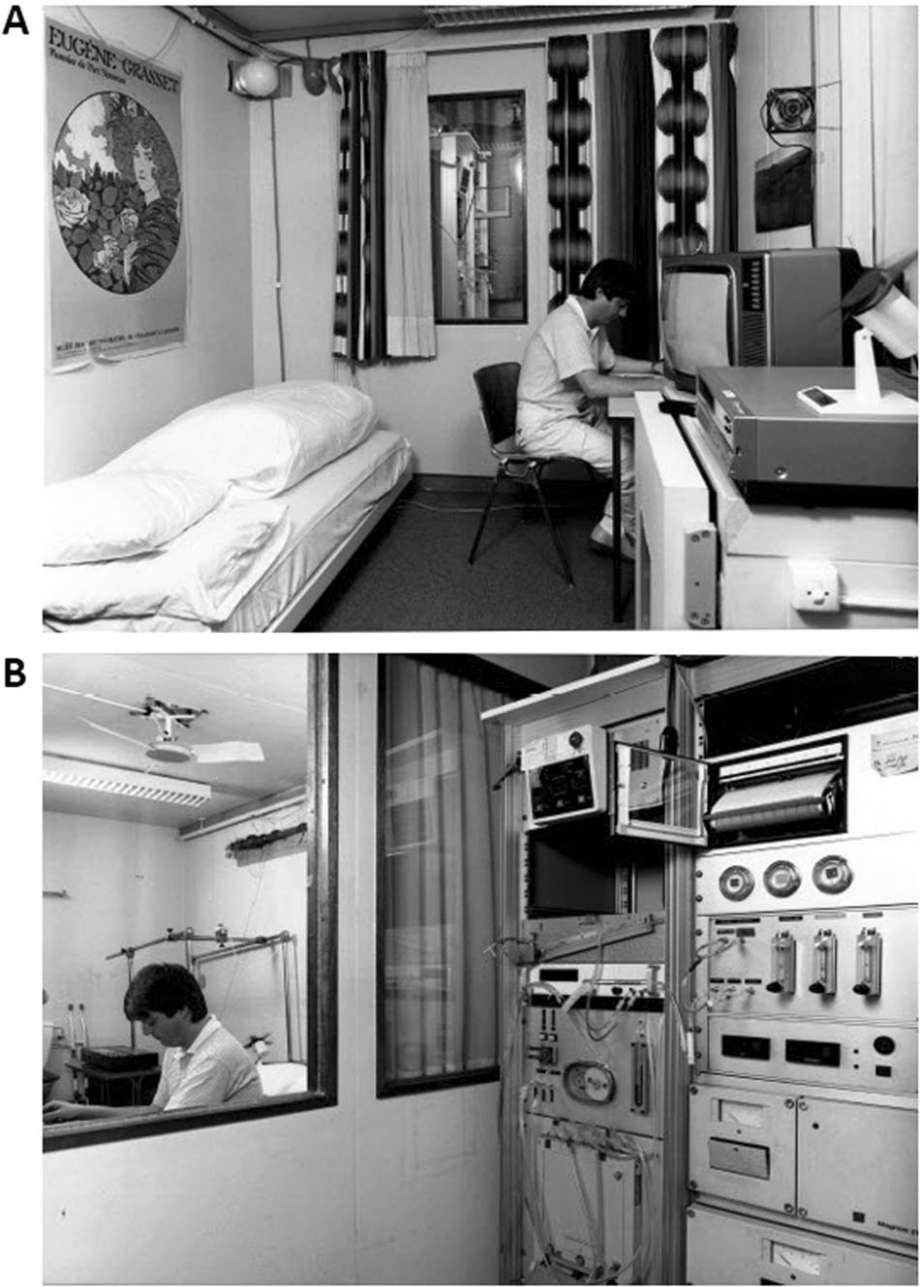
The Lausanne Respiratory chamber (used from 1980 on) is an open calorimetric system of the “pull” type (air pulled out of the chamber). **a** Inside view of the respiratory chamber (31 m^3^) with studio-like furniture. **b** View from outside with control units. Technical details are given in Ravussin et al. [46]

By late 1970s, it became clear that longer periods of measurements of EE and substrate oxidation were required to better mimic the 24-h life cycle incorporating the various components of daily EE, that is, metabolic rate at rest while awake and during sleep, the overall thermic effect of daily meals, as well as spontaneous physical activity (SPA). Evidently, this could not be achieved by a ventilated hood system nor with the small size direct calorimetric chamber used in Lausanne (1.56 m^3^) with the subject sitting on a chair (see Fig. 6). The construction of larger indirect calorimetric (respiratory) chamber capable of assessing energy metabolism over a day or more was thus essential in advancing knowledge in the field of energy balance and obesity (Fig. 7). Such a studio-size chamber—furnished with desk, chair, table, fold-out sofa bed and equipped with radio and TV set, sink, toilet and a radar to detect movement—would thus offer many advantages. Although the specific components of EE are not measured separately in the respiratory chamber, some of them can be individually identified and analyzed over specific time intervals, for instance, during sleep, rest, activity, meals, or over 24 h. The Lausanne respiratory chamber [46] played a central role in evaluating fundamental aspects of energy balance and macronutrient balance, which in turn form the basis of a better understanding of obesity and metabolic factors that contribute to its development. In particular:

- It established that the obese people have higher daily EE than lean people, a difference that is largely accounted for by their higher resting metabolic rate (in turn due to their higher lean body mass) and to a minor extent by an increased cost of moving the extra weight of the obese [46, 47]. An innovative low-cost method was used to track inconspicuously the SPA in the chamber. This was based on a fixed radar system [48]. By combining SPA with EE (measured at 15-min intervals over 24 h) it was possible to calculate at zero activity level the average overall thermogenic responses of the three meals served per day [49].
- It provided evidence that, in some obese individuals, a defective thermogenic response to food (in part due to insulin resistance and/or a blunted response of the sympathetic nervous system) is a factor in the pathogenesis of obesity, and that after weight loss, diminished diet-induced thermogenesis, together with metabolic adaptations in other components of daily EE, contributes to the relapse of obesity [37, 50].
- It demonstrated, through a series of studies conducted in collaboration with Jean-Pierre Flatt that (i) the capacity of de novo lipogenesis (i.e., synthesis of fat from non-lipid sources) was low in humans (lean or obese) in eucaloric conditions [51], thereby underscoring the fact that carbohydrates are not easily converted to fat. The exception is when massive carbohydrate overfeeding for more than a week was fed to normal weight subjects [52]. And that (ii) after ingesting a high-fat load, post-ingestive fuel selection favors the oxidation of dietary proteins and carbohydrates but not fat [53]. This demonstration of the failure of dietary fat intake to promote fat oxidation, thereby leading to dietary fats being preferentially stored as triacylglycerol in adipose tissue, is now considered as an important major factor favouring the development of obesity. A comprehensive review on the assessment of EE and fuel utilization in humans using direct and indirect calorimetry was published in 1987 [54].
- It served to quantify the impact of “drugs of everyday life” (caffeine [55], nicotine [56] and alcohol [57]) on daily EE and substrate oxidation, with implications for the weight gain that follows cessation of cigarette smoking (nicotine being a potent stimulus of sympathetic control of thermogenesis) and that alcohol consumption by inhibiting fat oxidation indirectly favors the storage of dietary fats, and hence promoting obesity.
- It provided the initial validation of the doubly labeled water isotopic technique as a method for assessing total EE over weeks in humans [58], which is now widely used in many parts of the world to assess free-living EE and hence provides a better estimate of energy requirements.

### The Lausanne respiratory chamber as a model for other chambers

**Fig. 8:**
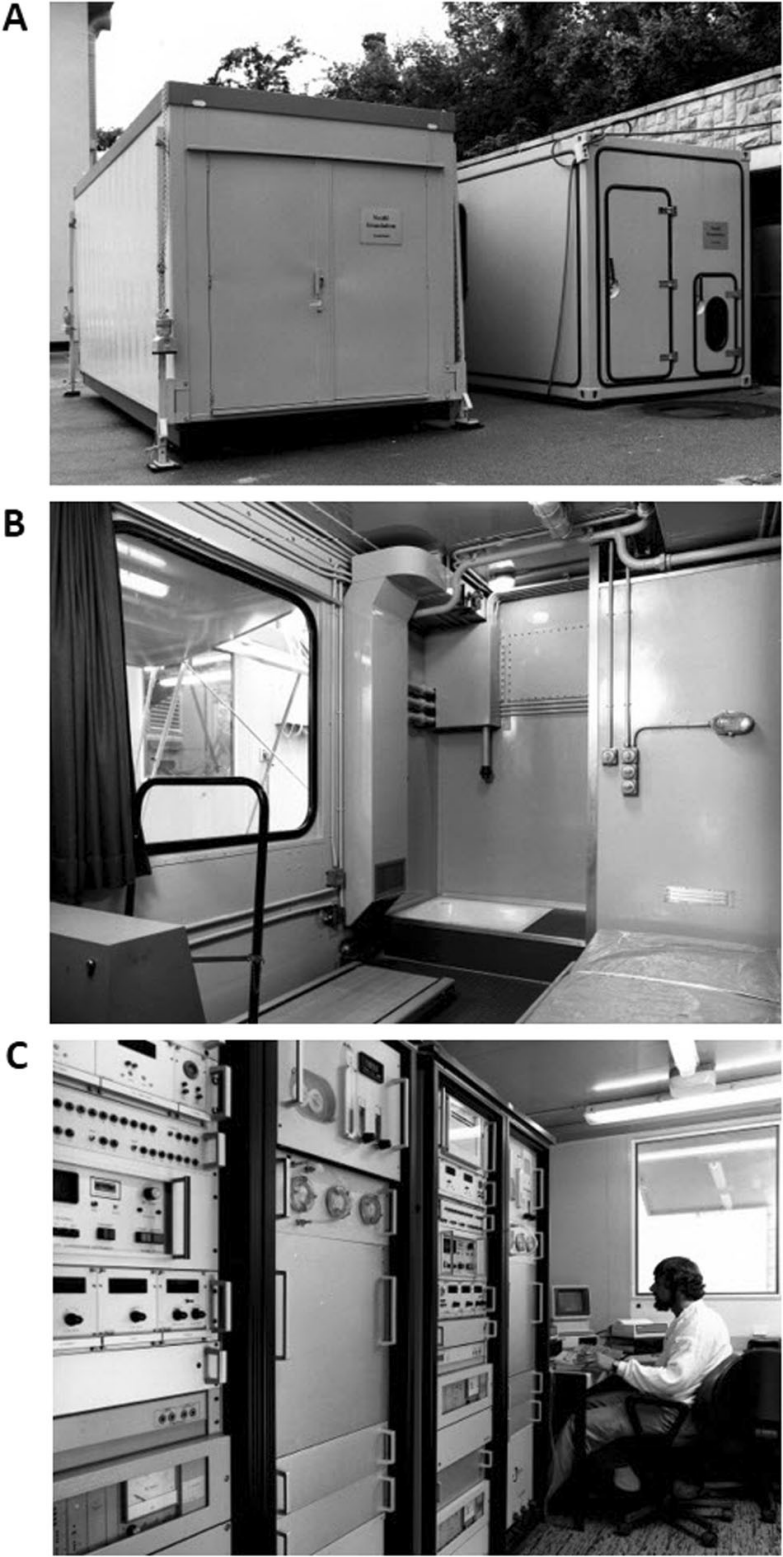
The Gambia (Africa) Respiratory chamber (build in 1985). It is an open calorimetric system of the “push” type (air pushed into the chamber). **a** Outside view of the control units (left) and of the respiratory chamber (right). The box was specially constructed according to military standards to withstand extreme changes in temperature. **b** Inside view of the respiratory chamber (27 m^3^) with bed, Turkish toilet and treadmill. **c**. Control units with duplication of all instruments (as backup in case of technical failure). Technical details are given in Charbonnier et al. [60]

In the decade following its construction and operation, the Lausanne respiratory chamber served as a model for other indirect calorimeter chambers constructed in other parts of the world (involving various expatriate Swiss researchers), in particular:

(i) The respiratory chamber of the NIH branch in Phoenix, Arizona [59]—for metabolic phenotyping of the Pima Indians, an American Indian community whose high prevalence of obesity and type 2 diabetes might be the consequence of a “thrifty genotype”.
(ii) A transportable whole-body indirect calorimeter designed for use in the tropics [60] was built in Lausanne as part of a joint project between the Universities of Lausanne-CH and Cambridge-UK for studies in villages of The Gambia (where ambient temperatures could range from 16 to 44 °C and dewpoints from −8 to 24 °C). This calorimeter (Fig. 8) was built to study EE of people having chronically or acutely low levels of food intake and to investigate energy adaptations made by individuals during periods of limited food intake. These studies revealed various energy sparing strategies (through reductions in various compartments of daily EE), which allow this population to cope with a marginal level of dietary intake during the hungry season [61, 62], including energy sparing strategies by pregnant women to protect fetal growth [63].

### “Bedside” calorimetry for clinical nutritional assessment

The advent of commercial metabolic carts (in the 1980s) bringing ventilated hood indirect calorimetry to the bedside laid down the foundations for the School of Lausanne to be at the forefront of research into the energy metabolism of acutely ill patients—who are often in a state of hypermetabolism and protein–calorie malnutrition). In collaboration with René Chiolero at the CHUV (Centre hospitalier universitaire vaudois, University Hospital of Lausanne/Vaud), acute indirect calorimetry studies in Lausanne evaluated the energy and nutrient needs to avoid tissue depletion in a variety of critically ill conditions, including in patients after surgery in oropharyngeal cancer, patients with liver cirrhosis, elderly patients after surgery (femoral neck fracture), patients with severe head injury and those with hypermetabolic response to burn injury [64–67].

The School of Lausanne, in collaboration with Jean-Léopold Micheli at the CHUV, also provided important insights into energy and protein metabolism of the neonates by applying “bedside” investigative methods that coupled indirect calorimetry with nutritional balance and stable (non-radioactive) isotopic techniques [68]. Their investigations about the postnatal time course of heat production and thermogenic effect of feeding [69], the metabolic cost of growth in premature babies in Lausanne [70] and the energetic cost of growth in “normal” infants living in Africa (The Gambia) [71], as well as the metabolic cost of whole-body protein synthesis and turnover using N15 Glycine [72, 73], have been key contributions toward understanding the physiological aspects of energy and protein metabolism directly related to the extrauterine adaptation, with implications for helping every day’s neonatal practice [68].

## The school of Geneva

### Mechanisms of organ/tissue thermogenesis: the need for microcalorimeters

In the early 1970s, while a research focus in Lausanne was about thermal regulation in the human neonate [27], Lucien Girardier and his collaborators in Geneva were extending their research in the electrophysiology of cardiac and skeletal muscle to that of BAT [74]. This was at a time when tissue’s high capacity for heat production in response to cold was already well established in small mammals (including the human neonate), but little was known about the underlying physiological and biochemical effector mechanisms. It was also an era of considerable controversies about the energy cost of cellular ionic homeostasis of central importance for cellular maintenance and functions, namely the two primary-active transport processes, Na-K transport across plasma(sarco)lemma and Ca^2+^ transport across the endo(sarco)plasmic-reticular membrane [75].

Based upon their initial observations [74] that the membrane potential recorded in isolated segments of rat interscapular BAT was markedly reduced by catecholamines, hypoxia and cooling of the tissue, the Geneva researchers suggested that changes in ionic permeability of the membrane and in the rate of active Na-K transport (sodium pumping) might be contributing factors in the regulation of heat production in BAT. In order to obtain quantitative estimates of the cellular energy requirement associated with this ionic transport process in BAT, as well as in skeletal muscle, they went on to construct a direct microcalorimeter that could measure the *time course* of heat production rate in these tissues.

### The Geneva microcalorimeters

**Fig. 9:**
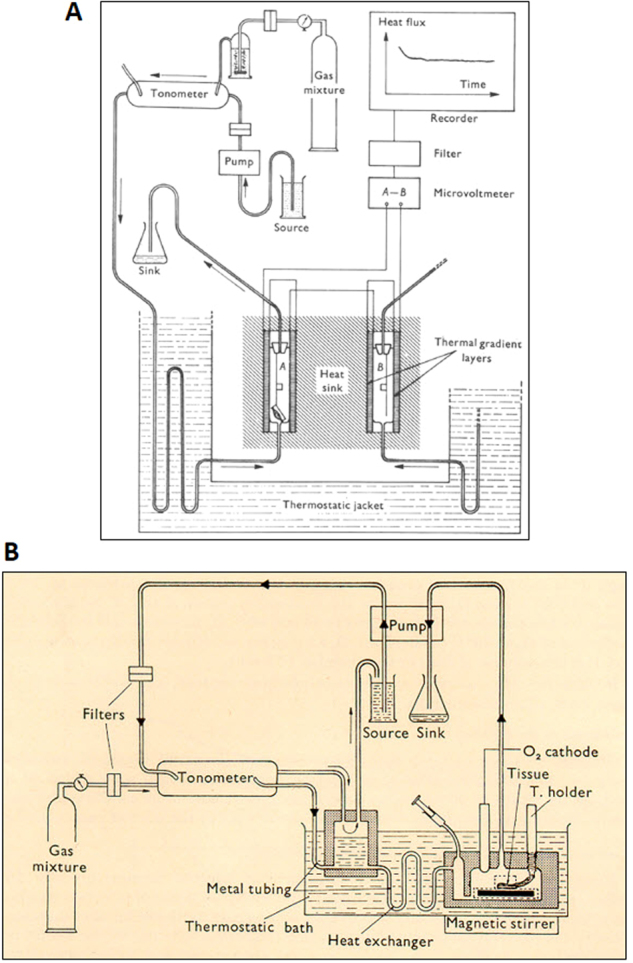
**a** Direct microcalorimetry for determination of energy expenditure in isolated tissues (rat and mice isolated muscles, BAT). The experimental setup is discussed in Chinet et al. [76]. **b** Indirect microcalorimetry by repeated O_2_ uptake determinations in tissues ex vivo. The system can be used for 4–6 h in isolated muscle or in BAT with its own innervation. The experimental setup is discussed in Barde et al. [77]

This direct microcalorimeter [76], which was designed in collaboration with George Spinnler, was a thermic flux apparatus, which operated on the principle that the voltage difference between two series of gradient layers (test chamber minus symmetrical control chamber) is proportional to the tissue heat production rate (Fig. 9a). The major advantage of this microcalorimeter was that it allows the detection of small changes developing within minutes. The rate of heat production could also be directly compared with the rate of O_2_ consumption determined by measuring the O_2_ uptake in the contralateral muscle (or another BAT fragment) using an O_2_ cathode in a bubble-free liquid phase. The latter indirect microcalorimetry system (depicted in Fig. 9b) involving repeated O_2_ uptake determinations in the test chambers was developed a couple of years earlier in Geneva [77] with the objective of studying the “*neuro-adipose synapse*” in the control of BAT thermogenesis. In fact, this study [77] provided evidence that, under the ex vivo conditions, the nerve endings in BAT fragments retain their physical characteristics for several hours with the presence of a basal tonic release and reuptake of noradrenaline in the synaptic cleft.

### Microcalorimetry research outcome

Over the decade that followed their construction, these two microcalorimeters in Geneva have been at the center of investigations that have provided important insights into the sympathetic neural control of BAT thermogenesis [78, 79] and the role of ion cycling [76, 80–82] and substrate cycling [83] in skeletal muscle heat production—with implications for their perturbations in a variety of pathological conditions that include thyroid disorders, obesity, diabetes, skeletal muscle lipotoxicity and Duchenne muscular dystrophy. Furthermore, the application of these novel microcalorimetry techniques to study BAT metabolism in rats with specific brain lesions [84–86] and in animal models with hormonal disorders resulting in defects either in cold-induced thermogenesis, diet-induced thermogenesis or in both [87–90] led to development of an “operational model of the regulation of BAT thermogenesis” with anatomical clues concerning pathways connecting thermal and weight regulations [90]. Many facets of this operational model are still valid to-day, and reflect the outcome of considerable research conducted by Lucien Girardier, Josiane Seydoux and Auguste Chinet, often in close collaboration with other Geneva University laboratories directed by Bernard Jeanrenaud, Albert Burger and Jean-Paul Giacobino.

### From micro- to macro-calorimetry

By early 1990s, the laboratories in Geneva had also constructed whole-body indirect calorimeters (one for the rat and one for humans), whose use have generated novel concepts in the field of energy metabolism and obesity, as outlined below.

#### Quantification of behavioral regulation of thermogenesis

**Fig. 10:**
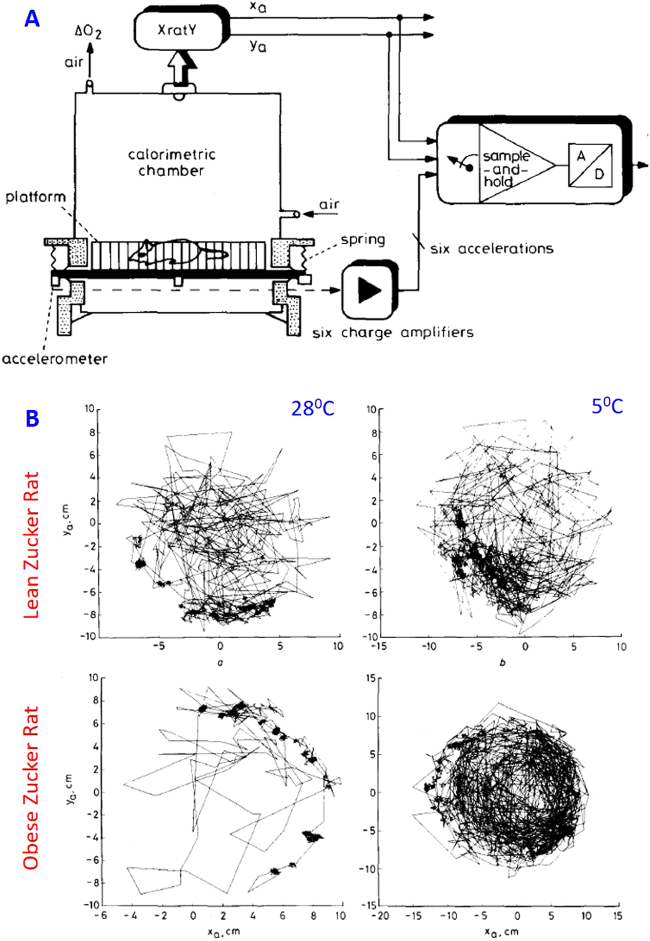
The ERGORAT for quantifying cost of physical activity. **a** Diagram of the system with the indirect calorimetry chamber, *X*/*Y* axis rat location by infrared LED, and external work assessment by accelerometry. **b** Distances covered by a lean and an obese Zucker rat during the control period (28 °C) and during cold exposure (5 °C). Panels **a** and **b** are drawn from Aminian et al. [91]

The Geneva researchers (in collaboration with engineers in EPFL) constructed of a real-time ergometric system (ERGORAT, Fig. 10a) for measuring EE due to muscular activity of small mammals [91]. It consisted of an open-circuit metabolic chamber supplemented with an ultrasensitive ergometric platform equipped with six unidirectional accelerometers to detect all the vibrations produced by a freely moving rat to a platform on which it is living, as well as with an opto-electronic device for location of the rat’s center of mass. The values of the mechanical energy transferred to the platform could be computed every second.

Using this system, they quantified the energy cost of the low level of the physical activity of freely moving rats, and showed that when exposed to acute cold, the obese Zucker rats with dysfunctional BAT were able to increase their metabolic rate to the same extent as their lean counterparts by increasing their locomotor activity [91, 92]. In these obese animals, SPA showed features of behavioral regulation (Fig. 10b), whereas in the lean rats such features were obscured by the involvement of alternative thermoeffectors (presumably BAT), which contributed to >60% of the increase in EE.

These studies, while emphasizing the high energy cost of low speed locomotion (such as that observed during spontaneous activity of the rat), also revealed that activity-associated thermogenesis can be conceptualized as a quantitatively important component of the thermoregulatory behavior. Indeed, the demonstrations in humans that variability in thermogenesis also resides in SPA [93] underscore the potential importance of SPA as a regulated behavioral component of EE of relevance to human weight regulation and predisposition to obesity.

#### Targeting the neuro-adipose synapse with bioactive food ingredients

The need for translational science occurred following microcalorimetry studies in BAT demonstrating that sympathetic control of thermogenesis could be potentiated by interference with negative modulators of noradrenaline in the *neuro-adipose synapse* with bioactive ingredients present in green tea—namely caffeine and catechin-polyphenols [94]. The newly constructed human indirect calorimeter chamber in Geneva provided the proof-of-concept for enhancing sympathetic control of thermogenesis and fat oxidation with green tea extracts in lean and overweight human subjects [95], and triggered considerable research interest in the potential role for polyphenols in the management of obesity [96].

## The school of Zurich

Since the early days with Max Kleiber, the Swiss Federal Institute of Technology in Zurich (ETHZ, Department of Animal Sciences) has mostly been interested in farm and domestic animals to determine the quantitative nutritional requirements of domestic animals (such as cats, pigs, cows and steers) depending upon sex, physiological state (growth, gestation and lactation) and a variety of environmental factors. Numerous studies were realized under the guidance of Alfred Schürch (1916–1998) and later of Caspar Wenk. The Department developed half a century ago [97] homemade respiration calorimeters, tailored for middle size animals (typically pigs), allowing to perform accurate 24-h EE and energy balance studies under confined and controlled conditions. In addition, total Nitrogen balance was coupled to energy balance measurements and body composition. Measurements of metabolizable energy and protein intakes, at different levels of dietary fibers, were also performed.

The effect of deliberately feeding excess energy, respectively, protein (or both together) on whole-body energy metabolism and changes in body composition of the animals could be analyzed. The purpose was to optimize the energy and protein levels in order to achieve a theoretical maximum energy/protein retention, without excessive fat storage in the animals, as well as to calculate the net efficiency of energy utilization for protein retention versus fat retention. Optimizing financial cost of feeding with maximum protein gain and minimum fat deposition is important considering that, already at that time, the demand of the consumer for lean meat was progressively increasing.

## Back to the future

The earlier studies of Max Kleiber to compare nutrient requirements of various animals, as well as the food rationing studies by Alfred Fleisch during the last World War characterized by post-war hyperphagia and body weight overshoot have generated a strong interest in Switzerland for human and animal metabolic studies. In the decades following war and with the emergence of diseases of affluence, it became clear that assessment of EE was of central importance for understanding how body weight is regulated. This period has seen the emergence of various research groups in Switzerland, developing tools and concepts that will serve as a strong basis for later research in the fields of obesity, anorexia, fat overshoot during catch-up growth and weight cycling. Many issues remain to be addressed, but thanks to our predecessors, we are a little bit less ignorant.

This review started with the description by Konrad Gessner in 1551 of a tissue that was judged as neither fat, nor flesh and which turned out to be BAT. However, up to the turn of this century, the presence of BAT in humans was thought to be limited to newborns, where it serves for their successful defense of body temperature as newborns have not yet developed shivering capacity [98]. By an interesting twist in history, the first article describing the presence of BAT in adult humans can be traced back to Zurich in 2002 [99]. This is perhaps the start of a foreseeable future where one might expect nuclear medicine and nano-technologies to provide non-invasive tools to assess organ and tissue-specific metabolism, as well as their interactions. Whether or not Swiss scientists will lead this research remains to be seen, but regardless, such development would no doubt revolutionize our understanding of energy metabolism in health and disease.
